# HPA axis function during adjunctive high-dose dexamethasone use in chemotherapy: a prospective pilot study

**DOI:** 10.3389/fendo.2026.1810229

**Published:** 2026-04-23

**Authors:** Elżbieta Turska, Krzysztof Lewandowski, Igor Symonowicz, Magdalena Kobus, Wojciech Horzelski, Tomasz Kubiatowski, Justyna Marchewka-Długońska, Ewa Kalinka

**Affiliations:** 1 Department of Oncology, Polish Mother’s Memorial Hospital – Research Institute, Lodz, Poland; 2 Department of Endocrinology & Diabetes, Collegium Medicum Mazovian University of Plock, Plock, Poland; 3 Institute of Biological Sciences, Faculty of Biology and Environmental Sciences, Cardinal Stefan Wyszynski University in Warsaw, Warsaw, Poland; 4 Oncology Clinical Trials Support Centre, Polish Mother’s Memorial Hospital – Research Institute, Lodz, Poland; 5 Department of Applied Computer Science, Faculty of Mathematics and Computer Science, University of Lodz, Lodz, Poland; 6 Oncology Clinic with a Gynecological Oncology Subdivision of the University Hospital in Rzeszow, Rzeszow, Poland; 7 Department of Oncology, Radiotherapy and Translational Medicine, Rzeszow University, Lodz, Poland

**Keywords:** adrenal insufficiency, breast cancer, chemotherapy, dexamethasone, HPA axis

## Abstract

**Introduction:**

Glucocorticoid-induced adrenal insufficiency is a well-known adverse effect of glucocorticoid therapy, occurring not only with oral administration but also with intramuscular, inhaled, and other routes of administration. However, the impact of intermittent high-dose glucocorticoids used during chemotherapy on hypothalamic–pituitary–adrenal (HPA) axis function remains incompletely understood.

**Methods:**

In this prospective pilot study, 47 women with breast cancer receiving paclitaxel-based chemotherapy with dexamethasone premedication were evaluated. Morning serum cortisol and adrenocorticotropic hormone (ACTH) concentrations were measured before dexamethasone administration at baseline and prior to subsequent weekly chemotherapy cycles. Longitudinal trends in cortisol and ACTH concentrations were analyzed using ANOVA and Spearman correlation.

**Results:**

Across the entire group, morning cortisol and ACTH concentrations showed an overall downward tendency during treatment. However, no statistically significant changes were observed. The results did not demonstrate significant suppression of morning cortisol or ACTH concentrations during intermittent high-dose dexamethasone administration in this group.

**Discussion:**

These findings underline the limitations of single cortisol measurements and highlight the need for a longitudinal approach when assessing adrenal function in oncology patients. Further studies incorporating dynamic testing are required to determine the optimal method for evaluating HPA axis function during intermittent glucocorticoid exposure.

## 1 Introduction

Glucocorticoids are known for their anti-inflammatory and immunosuppressive effects. They are used for many conditions, but systemic therapy can suppress the hypothalamic–pituitary–adrenal (HPA) axis, causing iatrogenic adrenal insufficiency (AI). This tertiary AI results from decreased corticotropin-releasing hormone (CRH) and adrenocorticotropic hormone (ACTH), leading to inadequate cortisol secretion, especially during stress, or intercurrent illness, posing the risk of life-threatening adrenal crisis (1), thus, underscoring the importance of prompt diagnosis. Despite widespread use, there are gaps in quantifying the prevalence of glucocorticoid-induced adrenal insufficiency (GIAI), notably in oncology settings where high-dose dexamethasone treatment is frequently employed (1, 2).

Evidence from the past decade indicates that suppression of the HPA axis (Hypothalamic–pituitary–adrenal axis) is common across various glucocorticoid regimens. Joseph et al. (2) found that glucocorticoid-induced adrenal insufficiency occurs in many clinical situations, with incidence rates varying by dosage, duration, and formulation. Suppression can happen even with short-term or intermittent use, challenging earlier beliefs that only long-term daily use is risky. Broersen et al. (3) noted that up to 27% of patients treated with systemic glucocorticoids show biochemical signs of AI, and 48% display insufficiency after treatment cessation, thus indicating that recovery may be slow and unpredictable. Such findings highlight the fact adrenal insufficiency is often underdiagnosed due to nonspecific symptoms and limitations of morning cortisol as a diagnostic tool (3).

Recent updates to clinical recommendations point to growing awareness of this issue. The 2024 joint guideline from the European Society of Endocrinology and Endocrine Society (4) offers a detailed framework for diagnosing and managing glucocorticoid-induced adrenal insufficiency. These guidelines emphasize that dexamethasone, due to its high affinity for the glucocorticoid receptor, long biological half-life, and lack of mineralocorticoid activity, has one of the highest potentials for HPA axis suppression among glucocorticoids. However, despite these theoretical and mechanistic risks, the guideline authors note that important clinical situations—such as intermittent high-dose dexamethasone use in chemotherapy—are still not well studied (4).

This risk is especially significant for oncology patients, where symptoms of adrenal insufficiency—such as fatigue, nausea, hypotension, anorexia, and overall weakness—closely resemble common chemotherapy side effects. Carelle et al. (5) demonstrated that these symptoms are both common and frequently underreported by cancer patients, which can mask adrenal insufficiency and delay diagnosis. If adrenal suppression goes unrecognized, patients may face serious dangers during other illnesses, surgeries, or dehydration episodes, as their body’s cortisol needs might exceed their suppressed production (5).

Assessing adrenal function biochemically is challenging. Morning serum cortisol levels are influenced by circadian rhythms, changes in CBG (Corticosteroid-Binding Globulin), liver conditions, and intercurrent illness (1). Liver stress from chemotherapy can alter CBG, complicating cortisol interpretation. Dynamic testing with synthetic ACTH (Synacthen^®^) remains the gold standard, but standard cut-offs may not suit patients on intermittent high-dose dexamethasone. Broersen et al. (3) and the 2024 guideline note the lack of validated cortisol thresholds for these patients (3, 4).

Overall, current evidence highlights the urgent need to systematically evaluate the risk of iatrogenic adrenal insufficiency in patients undergoing dexamethasone-supported chemotherapy. Identifying whether these regimens lead to clinically significant HPA axis suppression, determining the prevalence of lab-confirmed adrenal insufficiency, and establishing practical morning cortisol cut-off points are crucial steps to improve patient safety. Since dexamethasone is widely used in standard chemotherapy treatments, particularly in breast cancer, clarifying these risks is highly relevant for millions of patients worldwide.

In the present study, we aimed to evaluate longitudinal changes in morning cortisol and ACTH concentrations in breast cancer patients receiving dexamethasone as chemotherapy premedication, in order to assess potential suppression of the HPA axis.

## 2 Materials and methods

### 2.1 Participants

A group of patients with planned treatment of breast cancer was recruited at the Oncology Clinic of Mother’s Memorial Hospital. The study included 47 females, aged 33–81, with a median age of 59 years. The exclusion criteria were treatment with systemic steroids during the last 5 years and a history of adrenal failure or hypopituitarism. The Ethical Commission approved the study at the Polish Mother’s Memorial Hospital - Research Institute (no. 50/2021). Written informed consent was obtained from all participants after receiving detailed study information. **Table 1** presents descriptive statistics for age, body height, body weight, and BMI.

**Table 1:** Characteristics of the women in the study.

Parameter	Mean (± SD)	min-max
Age [years]	58.2 (13.83)	33.0-84.0
Body height [cm]	163.0 (5.96)	143.0-172.0
Body mass [kg]	72.8 (15.20)	49.0-114.0
BMI [kg/m^2^]	27.4 (5.63)	19.14 -42.91

### 2.2 Inclusion criteria and treatment protocol

The study enrolled female patients diagnosed with breast cancer who were eligible to receive sequential preoperative or postoperative chemotherapy. The study was designed as a prospective pilot study. The treatment regimen consisted of four cycles of AC chemotherapy (doxorubicin plus cyclophosphamide) administered every 14 or 21 days, followed by paclitaxel delivered either as monotherapy or in combination with carboplatin, for a total of 12 weekly doses. Premedication consisted of dexamethasone at a dose of 20 mg, administered twice before each paclitaxel infusion. Data collection (ACTH and cortisol levels) started with the first paclitaxel dose; measurements taken during AC chemotherapy were not included in the analysis. Blood samples were collected in the morning before dexamethasone administration.

### 2.3 Methods

Measurements of ACTH and cortisol levels were performed before administration of dexamethasone at 8:00 AM and repeated before subsequent weekly doses of dexamethasone. Subsequent measurements were performed after each weekly dose of dexamethasone (usually 20 mg). All patients underwent clinical evaluation prior to each chemotherapy cycle, including physical examination.

Cortisol and ACTH were measured at baseline and before each chemotherapy administration, both AC and paclitaxel. Cortisol and ACTH levels were assessed during the treatment phase in which paclitaxel chemotherapy was administered on a weekly schedule. In some cases, chemotherapy administration was delayed by one week due to abnormal laboratory results - such as neutropenia, thrombocytopenia, or symptomatic anemia - that precluded paclitaxel administration.

Prior to the weekly paclitaxel regimen, chemotherapy with doxorubicin and cyclophosphamide was administered every 14 or 21 days; however, during this phase a lower dose of dexamethasone (12 mg) was used as part of antiemetic prophylaxis.

Serum cortisol and plasma ACTH concentrations were measured using electrochemiluminescence immunoassay (ECLIA) on a Cobas Pro analyzer (Roche Diagnostics GmbH, Mannheim, Germany). Local laboratory reference intervals were 166−507 nmol/L for morning cortisol and 7.2–63.3 pg/mL for ACTH.

### 2.4 Statistical analysis

Statistical analysis was performed using Statistica 13.0 software. All calculations were carried out in spreadsheets after anonymization of the data. Initially, the average cortisol and ACTH levels, as well as the standard deviation and range of variability, were estimated. To compare the significance of differences in mean cortisol and ACTH levels in subsequent treatment cycles an ANOVA was used. The decision to use this test to assess the significance of differences in subsequent stages of treatment was made after checking compliance with the normal distribution using the Shapiro-Wilk test. To check the direction of the observed correlation and its statistical significance, Spearman’s rank correlation was used. Due to the number of patients examined, an *a priori* power test was performed, which for the one-way ANOVA used for comparisons with a sample size of 41 (measurement point 11) was 0.9612, indicating that with an assumed power level of 0.80, the results obtained can be considered accurate.

## 3 Results

**Tables 2**, **3** present the descriptive statistics for cortisol and ACTH levels at the various stages of treatment for the patients, with **Table 2** covering the entire study group and **Table 3** divided into two subgroups based on initial cortisol level. This allowed us to capture the general trends in cortisol and ACTH levels throughout the study (shown in **Figures 1**, **2**), as well as to record differences in patients who may have initially presented with adrenal dysfunction.

**Table 2:** Cortisol and ACTH level in specific time points during treatment with dexamethasone.

Time point	Cortisol [nmol/l]			ACTH [pg/ml]	
	n	Mean (± SD)	min-max	Mean (± SD)	min-max
1	47	349.34 (109.619)	148.99-659.40	17.25 (10.570)	2.60 -60.10
2	47	315.70 (98.745)	137.94-568.35	16.75 (9.480)	3.30-42.10
3	47	336.11 (104.543)	176.58-692.51	16.08 (7.495)	3.30-39.50
4	45	340.46 (117.765)	154.50-695.27	15.15 (8.023)	4.40-36.70
5	43	321.02 (130.692)	77.25-720.10	15.03 (7.373)	0.00-30.60
6	46	317.82 (117.328)	137.95-615.26	15.28 (14.094)	4.30-101.00
7	47	339.59 (173.604)	126.91-984.96	16.12 (8.55)	3.40-38.90
8	45	310.54 (110.539)	148.99-110.54	14.58 (7.359)	3.90-43.90
9	44	340.05 (166.21)	115.87-987.72	20.15 (29.767)	3.90-205.60
10	43	309.39 (126.712)	124.15- 626.29	14.22 (6.739)	3.70-34.30
11	41	302.95 (134.375)	118.64-855.29	15.85 (6.465)	4.10-37.50
p ANOVA		p = 0.7496 (MS = 11075; F = 0.673)		p = 0.6419 (MS = 125.4; F = 0.814)	
Spearman correlation		p = 0.1619 (r = -0.0628; r^2^ = 0.0039; t=-1.40)		p = 0.7056 (r = -0.0169; r^2^ = 0.0003; t=-0.37)	

**Table 3:** Cortisol and ACTH levels at subsequent stages of treatment, depending on baseline cortisol (measurement #1).

Time point	Cortisol/ACTH [pg/ml]	Baseline cortisol < 300 nmol/l			Baseline cortisol > 300 nmol/l		
		n	Mean (± SD)	min-max	n	Mean (± SD)	min-max
1	Cortisol	19	246.47 (41.448)	148.98-297.97	28	419.07 (82.774)	303.49-659.40
	ACTH		15.81 (8.998)	2.30-39.50		18.23 (11.573)	3.20-60.10
2	Cortisol	19	263.70 (73.761)	137.95-413.85	28	350.98 (115.72)	215.20-568.35
	ACTH		15.81 (9.00)	2.60-39.50		18.23 (11.573)	3.20-60.10
3	Cortisol	19	275.30 (69.744)	176,58-477.31	28	377.39 (104.921)	193.13-692.51
	ACTH		15.29 (8.258)	3.30-39.50		16.62 (7.036)	5.00-32.10
4	Cortisol	18	313.30 (115.643)	154.50-695.27	27	358.57 (117.791)	168.30-601.46
	ACTH		14.97 (7.578)	4.50-31.60		15.27 (8.447)	4.40-36.70
5	Cortisol	18	268.92 (103.122)	121.40-507.66	28	352.66 (137.062)	77.25-720.10
	ACTH		14.04 (6.930)	3.50-29.70		15.68 (7.700)	0.00-30.60
6	Cortisol	19	271.54 (95.633)	137.95-488.34	28	350.39 (121.767)	195.90-615.26
	ACTH		10.87 (4.233)	4.30-18.90		18.27 (17.423)	4.40-101.00
7	Cortisol	19	286.50 (103.809)	157.26-557.32	28	375.62 (202.072)	126.91-984.96
	ACTH		12.85 (6.220)	3.40-21.90		18.33 (9.290)	5.20-38.90
8	Cortisol	19	264.72 (70.996)	151.74-430.40	26	344.03 (122.941)	148.99-750.45
	ACTH		13.31 (6.065)	3.90-26.50		15.44 (8.115)	4.60-43.90
9	Cortisol	19	274.74 (60.128)	176.58-380.74	26	389.68 (202.074)	115.88-987.72
	ACTH		14.02 (9.646)	3.90-44.20		24.63 (37.987)	3.90-205.60
10	Cortisol	19	253.67 (75.336)	124.15-380.74	25	349.51 (141.679)	157.26-626.29
	ACTH		12.93 (5.102)	5.20-26.30		15.19 (7.717)	3.70-34.30
11	Cortisol	18	252.75 (76.096)	118.64-397.30	23	342.24 (157.101)	129.67-855.29
	ACTH		15.12 (5.399)	6.90-25.00		16.42 (7.258)	4.10-37.50
Cortisol p ANOVA			p = 0.5340 (MS = 6181; F = 0.901)		p = 0.6540 (MS = 15055; F = 0.774)		
ACTH p ANOVA			p = 0.6020 (MS = 43.55; F = 0.828)		p = 0.5621 (MS = 191.53; F = 0.870)		
Cortisol Spearman correlation			p = 0.6924 (r = 0.0278; r^2^ = 0.0008; t= - 0.396)		p = 0.2037 (r = -0.0745; r^2^ = 0.0055; t=-1.274)		
ACTH Spearman correlation			p = 0.1944 (r = -0.0912; r^2^ = 0.0083; t= - 1.321)		p = 0.8434 (r = -0.0116; r^2^ = 0.0001; t=0.198)		

**Figure 1:**
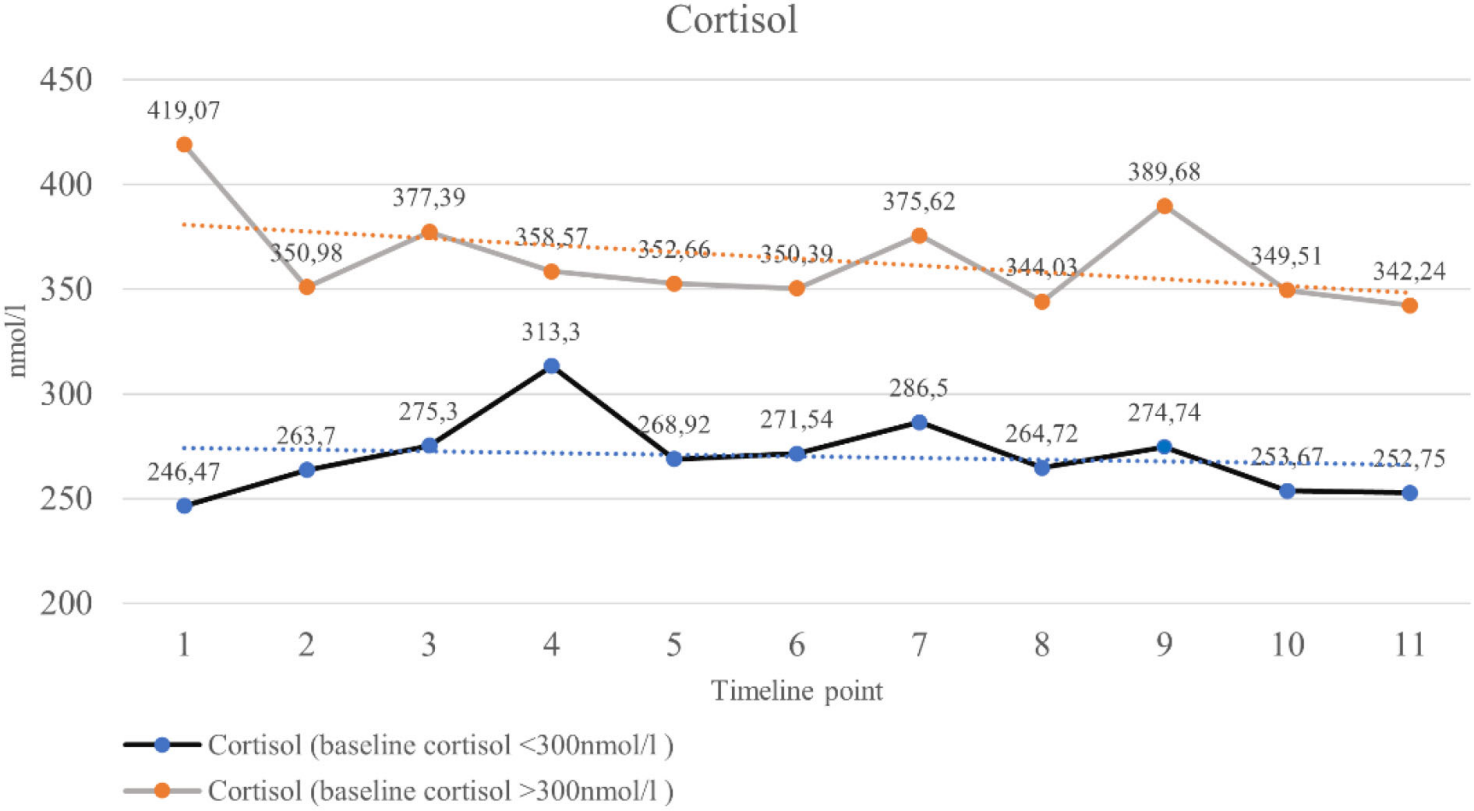
Changes in cortisol levels in the subsequent stages of the study.

**Figure 2:**
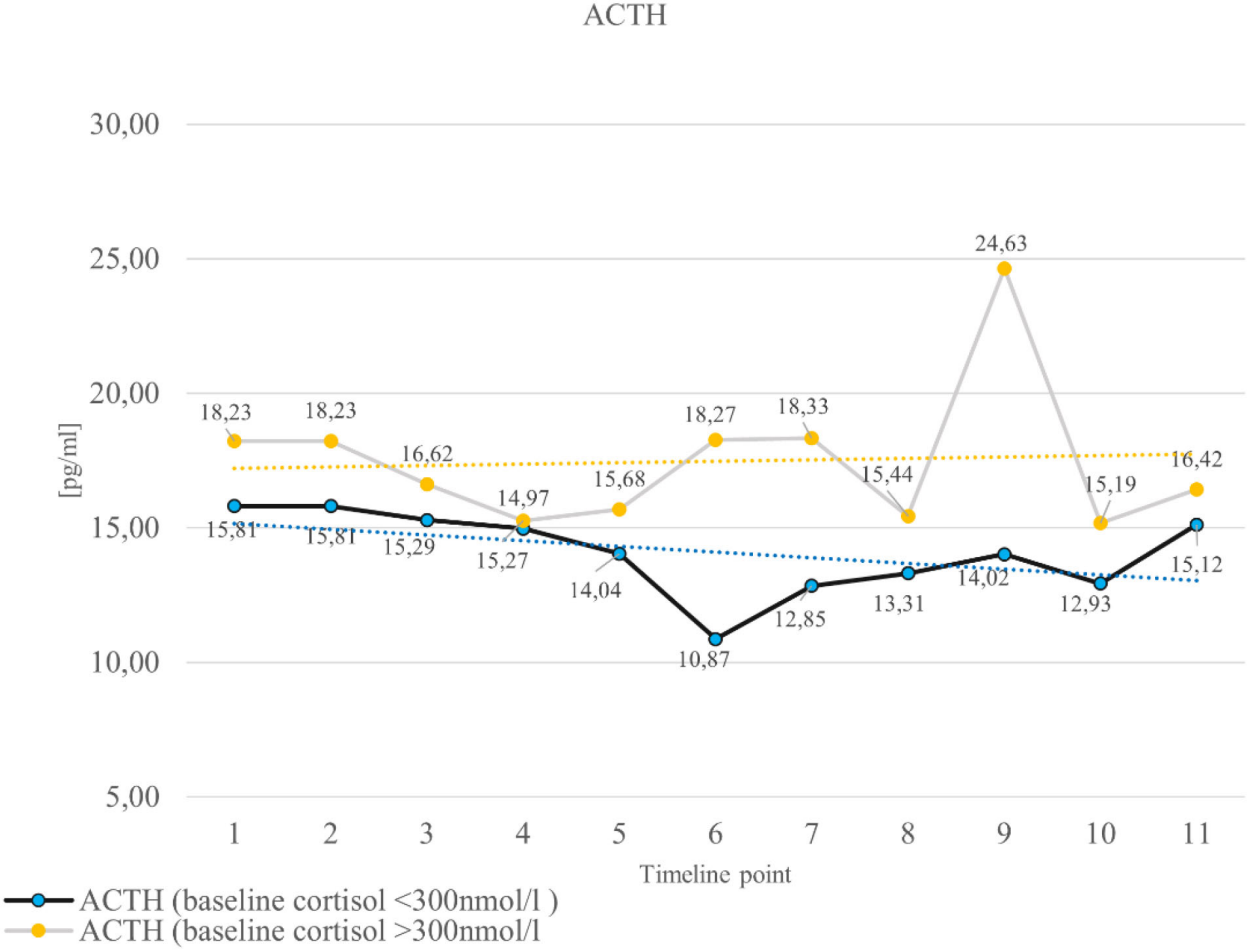
Changes in ACTH levels in the subsequent stages of the study.

### 3.1 Cortisol and ACTH dynamics in the entire group during treatment

**Table 2** shows the average cortisol and ACTH values for the subsequent stages of the study (11 measurement points). Generally, a downward trend in average cortisol measurements was observed in the subsequent stages of the study. This difference is especially noticeable between measurement 1 and measurement 11. In the case of these two measurements, the difference was 46.39 nmol/L. To compare the significance of differences in mean cortisol measurements across the individual groups, an ANOVA was used. However, the conducted significance testing did not indicate that the observed differences were statistically significant (p=0.8015). A correlation analysis was also conducted using Spearman’s rank correlation coefficient, which showed a downward trend (r = -0.066); however, these relationships were not statistically significant (p = 0.1289). An analogous downward trend in ACTH levels was observed. The exception was the ninth measurement, which showed an increase. ANOVA analysis indicates that the observed differences are not statistically significant, and correlation analysis confirms that changes in mean ACTH values across subsequent treatment stages are declining. In summary, these results indicate that the observed changes in cortisol and ACTH concentrations during treatment were not statistically significant.

### 3.2 Cortisol changes stratified by baseline cortisol level

The cortisol level in the first test would affect how it would change in the subsequent stages of treatment, and similarly, whether it would affect the ACTH level. Taking into account that Endocrine Society guidelines (4) stratify possible GIAI according to early morning cortisol concentrations into: adrenal insufficiency (cortisol <150 nmol/l, grey zone: cortisol 150–300 nmol/l, adrenal sufficiency for cortisol >300 nmol/l), we have divided patients into two groups according to early morning cortisol concentrations: (1) < 300 nmol/l and (2) > 300 nmol/l. This was done because in the group being studied, only one patient had cortisol below 150 nmol/l in the first test (the value obtained for this patient was 148.986 nmol/l). The results are presented in **Table 3**.

Among women with cortisol levels below 300 nmol/l in the first test, cortisol levels increased in subsequent stages of the test, while ACTH levels decreased. In both cases, the ANOVA test for significance did not indicate that the observed differences were statistically significant. Among women whose cortisol levels were above 300 nmol/l in the first test, both cortisol and ACTH levels decreased. Similarly, in the first group, the differences in the averages across the subsequent stages of the study were statistically insignificant.

## 4 Discussion

In this study, we evaluated the effect of adjunctive dexamethasone during chemotherapy on morning cortisol concentrations, which serves as an indicator of HPA axis suppression. These observations align with existing evidence that even brief courses or cyclical exposure to potent glucocorticoids, such as dexamethasone, may decrease endogenous cortisol production (2, 3).

Dexamethasone is widely used in oncology as an antiemetic, premedication for taxane therapies, and in various cytotoxic regimens. Breast cancer treatment often involves repeated high-dose dexamethasone cycles, totaling hundreds of milligrams over a short period. Although administered intermittently, these regimens can significantly suppress the HPA axis. Han et al. (6) first reported adrenal suppression in cancer patients receiving dexamethasone, demonstrating biochemical evidence even with short-term, cyclic use. This suggests that repeated high-dose pulses may have a suppressive effect comparable to or greater than chronic low-dose therapy, the primary risk for GIAI (1, 6).

Across the group, cortisol levels showed a downward trend from baseline to the final measurement; however, this change did not reach statistical significance. Considerable inter-individual variability in cortisol trajectories was observed. However, the marked inter-individual variability in our dataset underscores a key challenge emphasized in recent guidelines: susceptibility to (GIAI cannot be predicted solely by dose, duration, or frequency of glucocorticoid administration (4). Reviews examining adrenal suppression indicate that HPA axis suppression may happen even after short-term or low-dose glucocorticoid use. Additionally, the extent of axis suppression and recovery can vary widely between patients, underscoring that simple regimen metrics are not reliable predictors (7).

The most significant reductions in cortisol were observed in patients with initially high baseline levels (>300 nmol/L). This group showed a downward trend in cortisol levels, although the differences were not statistically significant. Conversely, patients with baseline cortisol levels <300 nmol/L (150–300 nmol/L) generally maintained stable levels. This suggests that isolated low and intermediate baseline cortisol levels cannot reliably predict subsequent adrenal suppression, particularly in the context of chemotherapy, where disruptions in circadian rhythm, systemic inflammation, and nutritional status may impact cortisol measurements (1, 6). The early treatment phase was characterized by a gradual decline in ACTH, consistent with the expected inhibitory effect of exogenous glucocorticoids on the HPA axis. Mid-treatment values showed greater fluctuation, including a pronounced peak at timepoint 9 attributable to a single outlier, which markedly increased variance. Excluding this extreme value, the mid-phase pattern remained generally stable without evidence of recovery of ACTH secretion. During the final phase of treatment, ACTH concentrations stabilized before decreasing again toward the end of therapy. Taking this into account, we need to underline, however, that so far, in contrast to cortisol, there are no normative data on ACTH concentrations with regard to specific cut-offs indicating adrenal insufficiency.

Morning cortisol levels show significant physiological variation influenced by circadian rhythm, stress, and assay differences, which limits the diagnostic usefulness of single measurements in intermediate ranges. From an endocrine standpoint, only clearly diminished early- morning cortisol levels provide strong biochemical evidence of adrenal insufficiency. In clinical practice, cortisol concentrations below about 5 µg/dL (140 nmol/L) are generally considered definitive for adrenal insufficiency, while higher levels typically require confirmation through dynamic testing, such as the cosyntropin stimulation test. These factors should be considered when interpreting borderline cortisol results (8). One of the most clinically relevant findings is the substantial number of patients who transiently had cortisol values <150 nmol/L at some point during their chemotherapy course. Although morning cortisol is widely used as an initial screening tool for adrenal insufficiency, its diagnostic accuracy is limited, especially under conditions that affect CBG, such as hepatic dysfunction and alterations in protein metabolism caused by chemotherapy (9, 10). Therefore, as highlighted in endocrinology guidelines, morning cortisol alone is insufficient to confirm or exclude GIAI (10, 11). The overall pattern may be compatible with transient pituitary suppression, although no statistically significant changes were demonstrated. These findings suggest that ACTH may have limited utility as a screening parameter in this clinical context and emphasize the need for integrated assessment using morning cortisol and, if indicated, dynamic stimulation testing in this clinical population. Dynamic testing with ACTH stimulation remains the standard of care, although the primary aim of the recent Guidelines (4) was to limit the widespread use of dynamic testing. Although the standard 250 µg short Synacthen^®^ test (SST) has been used in such settings and is reported to have 92% sensitivity, it uses a very high (supraphysiological) ACTH dose, which carries a risk of a false- positive pass, as reported before (12, 13). Furthermore, complete abandonment of such tests in these settings is debatable, given that subnormal responses were reported in 16% (56 out of 350) at three or six months after the start of first chemotherapy, where dexamethasone was used as an antiemetic in cancer patients receiving chemotherapy (14). Hence, in our opinion, the precise algorithm for possible application of dynamic tests remains to be clarified. We suggest, however, that if early- morning cortisol remains in the “grey zone” (150–300 nmol/L), it should be retested, for instance, after a 7-10-day period. If cortisol concentrations still remain in the “grey zone”, then a short Synacthen^®^ test should at least be considered, though administration of hydrocortisone with subsequent periodic retesting can also be fully acceptable. Such a rationale should consider the availability of medical resources, including the number, frequency, and cost of subsequent clinic visits.

Reference ranges for early morning cortisol are often arbitrary, while, in fact, the impact of stress response of serum cortisol concentrations is often underestimated by professionals outside the field of endocrinology. Cancer-related stress, systemic inflammation, and metabolic disturbances linked to malignancy and chemotherapy are well known to activate the HPA axis and increase basal cortisol secretion (15, 16). Patients diagnosed with cancer, who are about to start chemotherapy that is associated with a very wide range of adverse effects (apart from anxiety associated with cancer disease) are likely to display higher cortisol concentrations prior to the start of therapy. Such phenomenon was also described in children who demonstrated much lower diurnal cortisol concentrations soon after cardiac surgery in comparison to values observed immediately prior to surgery (17) This clearly demonstrates that the impact of individual stress and anxiety of cortisol secretion might be even greater than an impact of surgery for congenital heart disease per se.

Baseline morning cortisol levels in our group were relatively high, likely reflecting e.g. cancer-related stress. Importantly, elevated cortisol levels can mask subtle HPA axis suppression when relying solely on morning measurements, as normal or even high values do not exclude impaired adrenal function (11, 18). Moreover, morning cortisol levels are influenced by factors such as circadian rhythm, and changes in CBG, which could possibly influence their diagnostic value in oncology patients (11, 19). Previous studies indicate that adrenal suppression might be detectable through dynamic testing despite normal basal cortisol levels (15). Therefore, our findings support cautious interpretation of morning cortisol and emphasize the need for longitudinal or dynamic testing in this clinical setting (11, 18).

We also note that the heterogeneity of cortisol trajectories observed here aligns with previous evidence. Han et al. (5) showed that even short-term dexamethasone, used solely as an antiemetic, caused adrenal suppression in many cancer patients. Similar patterns have been observed in non-oncologic settings; for example, Woods et al. (19) reported frequent suppression among patients receiving inhaled glucocorticoids, emphasizing that cumulative exposure—even outside chronic systemic therapy—requires clinical vigilance. These findings are further supported by recent data published in Acta Haematologica, which demonstrated that adjunctive high-dose dexamethasone during chemotherapy is associated with a significant risk of transient adrenal suppression, even when used intermittently as antiemetic prophylaxis. The authors emphasized that cumulative glucocorticoid exposure during oncologic treatment may lead to clinically relevant HPA axis dysfunction, reinforcing the need for biochemical monitoring and individualized assessment strategies in cancer patients (20).

Our reclassification analyses further illustrate the dynamic nature of adrenal function during chemotherapy. Many patients shifted between cortisol categories over time, and nearly all reached cortisol concentrations >300 nmol/L at some point, reflecting reactive increases that may be triggered by treatment-related stress. Conversely, the lowest recorded cortisol levels placed one-third of patients temporarily in the <150 nmol/L category, suggesting at least episodic vulnerability of the HPA axis. These findings underscore the importance of timing when evaluating adrenal function in oncology patients, as both nadir and peak cortisol levels can offer clinically meaningful insights. They also support the growing consensus that context—particularly cumulative glucocorticoid exposure and concurrent systemic illness—must be considered rather than relying on single threshold measurements (2, 21, 22). Evidence from non-oncologic populations further supports the idea that cumulative glucocorticoid exposure may cause clinically significant HPA axis suppression even without continuous systemic therapy. For example, Woods et al. (20) showed that adrenal suppression is fairly common among patients using inhaled glucocorticoids, with morning cortisol levels serving as a useful screening method to identify those at risk. These findings highlight that GIAI can happen with different routes of administration and in various clinical situations, emphasizing the need to monitor adrenal function in patients who have repeated courses of glucocorticoids (23).

A significant clinical implication of these results is the prevention of adrenal crisis. Although adrenal crisis remains infrequent, its outcomes can be severe (7). In a prospective group study, Hahner et al. reported that stress-related triggers frequently precipitate adrenal crises, including in patients with secondary adrenal insufficiency, underscoring the importance of vigilance in high-risk populations such as oncology patients (7, 24). Oncology patients might be at greater risk because symptoms of adrenal insufficiency and chemotherapy toxicity often overlap, making early warning signs harder to detect. Because symptoms such as fatigue, nausea, and hypotension are nonspecific, biochemical assessment becomes even more crucial (6, 7). Additional evidence supporting altered adrenal function in oncology populations has been reported in prospective studies evaluating stress hormone responses during chemotherapy. In a study of pediatric oncology patients, Boekstegers et al. (25) demonstrated that cortisol responses during acute illness or treatment-related stress were frequently attenuated, suggesting that repeated glucocorticoid exposure and systemic illness may impair normal HPA axis reactivity. Although conducted in a pediatric population, these findings support the concept that oncologic treatment environments may predispose patients to transient or subclinical adrenal suppression (25).

Our findings support a practical approach endorsed by contemporary guidelines: routine morning cortisol monitoring at baseline, periodically during high-dose glucocorticoid exposure, and after treatment, followed by ACTH stimulation testing in selected patients with persistently low or declining cortisol concentrations (4).

### 4.1 Limitations

Limitation of our study is the lack of dynamic testing and long-term endocrine follow-up, as many patients continued their oncological care outside our department. Nevertheless, to our knowledge none of the patients from the studied group were subsequently referred to our endocrine unit with suspected adrenal insufficiency.

## Data Availability

The data is available only on the reasonable request. Requests to access the datasets should be directed to kobus.magdalena@gmail.com.
